# Supplementary material for: Hyperaccumulator Stanleya pinnata: In Situ Fitness in Relation to Tissue Selenium Concentration
Source: Plants (Basel). 2022 Mar 3;11(5):690. doi: 10.3390/plants11050690 (PMC8912631; doi:10.3390/plants11050690)
Supplement: Supplementary file 1 [file plants-11-00690-s001.zip › plants-1605903-supplementary.pdf]

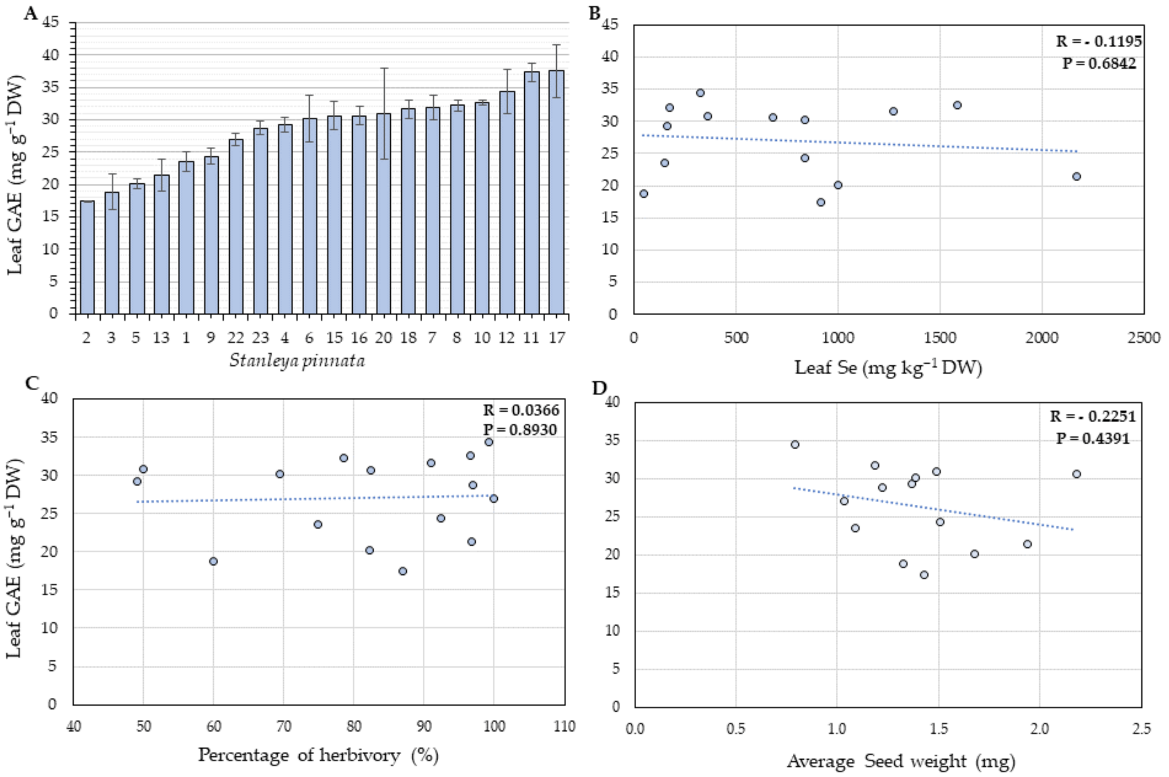

Supplemental Figure S1. A: Total phenolics concentration in leaves of *S. pinnata* plants growing at Coyote Ridge (locations indicated in Figure 2), expressed as gallic acid equivalents (GAE). (B-D) Correlation between leaf phenolics and leaf Se concentration (B), and number of leaves (C) and between seed phenolics concentration and average seed weight (D) . The numbers on the horizontal axis (A) represent individual plants at the locations indicated in Figures 2. Correlation coefficient R and P-value for panels B, C and D are shown in the graphs.

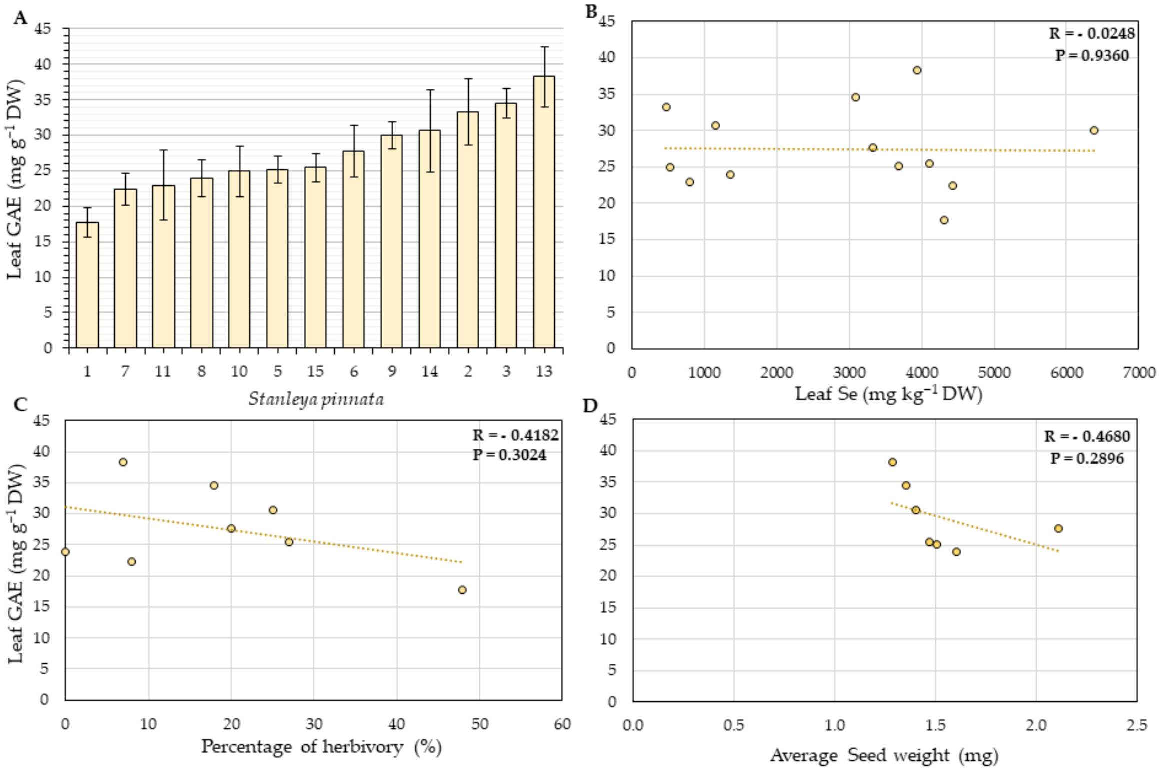

Supplemental Figure S2. A: Total phenolics concentration in leaves of *S. pinnata* plants growing at Pine Ridge (locations indicated in Figure 3), expressed as gallic acid equivalents (GAE). (B-D) Correlation between leaf phenolics and leaf Se concentration (B), and number of leaves (C) and between seed phenolics concentration and average seed weight (D) . he numbers on the horizontal axis (A) represent individual plants at the locations indicated in Figures 3. Correlation coefficient R and P-value for panels B, C and D are shown in the graphs.
